# Scientific Items

**Published:** 1881-07-20

**Authors:** 


### SCIENTIFIC ITEMS.

**How Long Man May Live.**—It was Professor Hufeland's opinion that the limit of possible human life might be set down at 200 years; and this on the general principle that the life of a creature is eight times the years of its period of growth. That which is quickly formed quickly perishes, and the earlier complete development is reached the sooner bodily decay ensues. More women reach old age than men, but more men attain remarkable longevity than women. Some animals grow to be very old. Horned animals live shorter lives than those without horns, fierce longer than timid, and amphibious longer than those which inhabit the air. The voracious pike exists, it is said, to an age of 150 years; the turtle is good for 100 years or more, and among birds the golden eagle is known to have lived nearly 200 years, while the sly and somber crow reaches the venerable age of a century. Passing up in the scale of life to man, and skipping the patriarchs, we find many recorded instances of longevity among the classic Greeks and Romans. Pliny notes that in the reign of the Emperor Vespasian, in the year 76, there were 124 men living in the limited area between the Appennines and the Po of 100 years and upward, three of whom were 140 and 4 over 135. Cicero's wife lived to the age of 163, and the Roman actress, Lucija, played in public as late as her 112th year. Coming down to more recent times, the most notable authentic instance of great age is that of Henry Jenkins, of Yorkshire, England, who died in 1670, 169 years old. He was a fisherman, and at the age of 100 years swam across rapid rivers. Another historic case is that of Thomas Parr, of Shropshire, a day laborer, who lived to the age of 152 years. When more than 120 he married his second wife, and till 130 he could swing the scythe and wield the flail with the best of his fellow-laborers. In his 152d year Parr went up to London to exhibit himself to the king. It proved an unlucky visit, for, violating the abstemious habit of a century and a half, the old man feasted so freely on the royal victuals that he soon died, merely of a plethora. On examination his internal organs proved to be in excellent condition, and there was no reason why he should not have lived much longer save for this unfortunate taste of royal hospitality. Professor Hufeland's roll of centenarians includes many more remarkable cases.

**The Process of Rumination.**—A sucking calf has a cud, or ruminates, very soon after its birth, but not for the first few days. The cud is the food which was last eaten; the cud comes only from the first stomach. The following description will explain the process: The cow's (and sheep's) stomach is in four compartments. The first, second and third are all open from the gullet, and are connected by a short canal called the esophagean canal. This is about four inches long. The passage from the third to the fourth stomach is from the further end of the third stomach or manifolds. When coarse food is eaten, or soft food or liquid is swallowed rapidly, it cannot pass through the canal fast enough, but forces open the valve or drops through the first opening and enters the first stomach or paunch. Here it is softened and macerated and made into a pulp. The cow, by some muscular arrangement, forces a portion of this food into the canal by which it is molded into a bolus, or long ball, and the reversed action of the gullet brings it to the mouth to be rechewed: this is the cud. When it is chewed the second time it is mixed copiously with saliva and becomes a thin liquid pulp, and, when swallowed, easily slides through the canal over the openings of the first stomach into the third, some coarser portions falling into the second stomach. When fine meal or pulpy food is swallowed it will pass the same way into the third stomach, where it is crushed in the manifolds, and goes into the last stomach, where alone it is digested. Whatever food goes into the third stomach cannot return to be ruminated. When a calf sucks the dam, the milk passing slowly, goes through the canal into the third stomach, and from that to the fourth, which is the rennet. When it is weaned and drinks from the pail, it swallows the milk so fast that some of it is forced into the first stomach, from which it is returned as a cud in the usual way. In the plan of exclusive meal-feeding in the winter, which was practiced by a few dairymen some time ago, the cows did not chew the cud at all until fed coarse fodder again.

**Value of the Dentaphone.**—Mr. Edmund Tribel, superintendent of the Royal Asylum for the Deaf and Dumb in Berlin, has made a series of extensive and critical experiments with the dentaphone as an aid to hearing, with entirely negative results. He states (Archives of Otology, December, 1880), that where deaf-mutes are concerned, the dentaphone, in its present condition at least, cannot be put to any practical use, not even as a means of advancing articulation, and he believes that the instrument cannot give any noteworthy assistance to any one whose hearing is in the least defective. These results agree entirely with those obtained by competent observers in this country. —*Medical News and Abstract.*

THE Nature says that an electric cable manufacturing firm in Neuchatel have made a highly important discovery in practical telegraphy. After a long and expensive series of experiments, they have succeeded in devising a method of laying cables, whereby the induction of the electric current from one wire to another, although the wires are in juxtaposition, is prevented. This discovery, of which no details are yet given, removes, it is assured, the last obstacle in the way of the widest possible extension of facilities for telegraphic communication.

IF a person of fair complexion expose himself to the electric light for some time in examining the action of lamps, the hands and cheeks will show all the symptoms of "sunburn," even in midwinter, and he will develop freckles on his countenance as quickly as when he goes about unprotected by a sun-umbrella in midsummer.

SPECIMENS of fossil woods and lignite are reported to have been brought to the surface from the depth of 191 feet while boring an artesian well at Galveston, Texas.

A MEMBER of the French Academy of Science has discovered well marked sexual differences in eels.

